# Consumption

**Published:** 1894-12-22

**Authors:** 


### SPECIAL HOSPITALS.

### CONSUMPTION.

**Brompton Hospital for Consumption and Diseases of the Chest**, S.W.—This hospital is well known as one where the patients are well cared for, and every effort is made to brighten their clouded lives. The maintenance of the new extension building entails an extra expenditure of several thousand pounds a year, and funds are urgently needed to meet this outlay. Many subscriptions have been withdrawn through failure of income and other causes; the committee are most anxious that their places should be filled up by other kind friends. Secretary, Mr. W. H. Theobald.

**City of London Hospital for Diseases of the Chest**, Victoria Park, E.—This hospital is situate in the East of London, where the diseases it treats are so common. About 1,000 in-patients and 15,000 out-patients are treated yearly. The average annual expenditure is more than £11,000, towards which only £370 of the income is assured. During the past three years the disbursements have exceeded the receipts by £6,500, and there is now a debt of £3,000 owing to the bankers in consequence of inadequate income during the current year. Assistance is much required. Donations or annual subscriptions will be thankfully received by the Secretary, Mr. T. Storrar-Smith, at the office, 24, Finsbury Circus; or by the bankers, Messrs. Barclay, Ransom, and Co.

**North London Hospital for Consumption**, Mount Vernon, Hampstead, N.W., and 41, Fitzroy Square W.—This institution was thoroughly reorganised and remodelled some years ago, and has now become one of the most useful of its class; 400 in-, and 3,000 out-patients are admitted each year. Secretary, Mr. Lionel F. Hill, M.A.; Matron, Miss K. Elphick.

**Royal Hospital for Diseases of the Chest**, City Road, E.C.—This is the oldest consumption hospital in Europe, having been founded by Her Majesty's father, the late Duke of Kent, in 1814. There are 80 beds, and last year 465 in-patients and 8,972 out-patients were treated. The expenditure exceeds £7,000, towards which there is an annual subscription list of £2,000, and dividends amounting to about £100. Two additional wards have recently been opened after having been closed for seven years, and funds are urgently needed. Donations will be gratefully acknowledged by the Secretary, or may be sent direct to the Chairman of the hospital, Mr. T. Andros de la Rue.

**The National Orthopædic Hospital**, Great Portland Street, though pursuing a very necessary and useful work, suffers from the failure of the public to understand its work and its needs. It seeks to restore the deformed to natural shape, and give new strength and vitality to those who are handicapped in the struggle for existence. The process of making the crooked straight and of setting the captives free is most often long and tedious, and the number of patients cannot therefore be compared with more general institutions. Some 1,400 in and out patients have been benefited in 1894. The hospital's pressing needs are first to see the end of a debt of £2,150 on the building, and to have its roll of annual subscribers increased to three times its present length. The beds are always occupied, and thirty patients accepted by the surgeons wait for admission the occurrence of vacancies. Secretary, Mr. A. J. Tresedder.

